# Functional Characterization of Six *SLCO1B1* (OATP1B1) Variants Observed in Finnish Individuals with a Psychotic Disorder

**DOI:** 10.1021/acs.molpharmaceut.2c00715

**Published:** 2023-02-13

**Authors:** Katja Häkkinen, Wilma Kiander, Heidi Kidron, Markku Lähteenvuo, Lea Urpa, Jonne Lintunen, Kati-Sisko Vellonen, Seppo Auriola, Minna Holm, Kaisla Lahdensuo, Olli Kampman, Erkki Isometsä, Tuula Kieseppä, Jouko Lönnqvist, Jaana Suvisaari, Jarmo Hietala, Jari Tiihonen, Aarno Palotie, Ari V. Ahola-Olli, Mikko Niemi

**Affiliations:** † Department of Forensic Psychiatry, Niuvanniemi Hospital, University of Eastern Finland, Kuopio FI-70240, Finland; ‡ Institute for Molecular Medicine Finland (FIMM), HiLIFE, University of Helsinki, Helsinki FI-00014, Finland; § Division of Pharmaceutical Biosciences, Faculty of Pharmacy, University of Helsinki, Helsinki FI-00014, Finland; ∥ School of Pharmacy, University of Eastern Finland, Kuopio FI-70211, Finland; ⊥ Mental Health Team, Finnish Institute for Health and Welfare, Helsinki FI-00271, Finland; # Mehiläinen Ltd, Helsinki FI-00260, Finland; ¶ Faculty of Medicine and Health Technology, Tampere University, Tampere FI-33100, Finland; ∇ Department of Psychiatry, Pirkanmaa Hospital District, Tampere FI-33521, Finland; ○ Department of Clinical Sciences (Psychiatry), Faculty of Medicine, Umeå University, Umeå SE-90187, Sweden; ⧫ Department of Psychiatry, University Hospital of Umeå, Umeå SE-90187, Sweden; †† Department of Clinical Medicine (Psychiatry), Faculty of Medicine, University of Turku, Turku FI-20014, Finland; ‡‡ Department of Psychiatry, The Wellbeing Services County of Ostrobothnia, Vaasa FI-65101, Finland; §§ Department of Psychiatry, University of Helsinki and Helsinki University Hospital, Helsinki FI-00014, Finland; ∥∥ Department of Psychiatry, University of Helsinki, Helsinki FI-00014, Finland; ⊥⊥ Department of Psychiatry, University of Turku and Turku University Hospital, Turku FI-20700, Finland; ## Department of Clinical Neuroscience, Karolinska Institutet, Stockholm SE-17177, Sweden; ¶¶ Center for Psychiatry Research, Stockholm City Council, Stockholm SE-11364, Sweden; ∇∇ Neuroscience Center, University of Helsinki, Helsinki FI-00014, Finland; ○○ The Stanley Center for Psychiatric Research and Program in Medical and Population Genetics, The Broad Institute of MIT and Harvard, Cambridge, Massachusetts 02142, United States; ⧫⧫ Analytic and Translational Genetics Unit, Department of Medicine, Department of Neurology and Department of Psychiatry, Massachusetts General Hospital, Boston MA-02114, United States; ††† Department of Internal Medicine, Satasairaala Hospital, Pori FI-28500, Finland; ‡‡‡ Department of Clinical Pharmacology, University of Helsinki, Helsinki FI-00014, Finland; §§§ Individualized Drug Therapy Research Program, Faculty of Medicine, University of Helsinki, Helsinki FI-00014, Finland; ∥∥∥ Department of Clinical Pharmacology, HUS Diagnostic Center, Helsinki University Hospital, Helsinki FI-00029, Finland

**Keywords:** pharmacogenetics, SLCO1B1, OATP1B1, organic anion transporting polypeptide 1B1, drug transporter

## Abstract

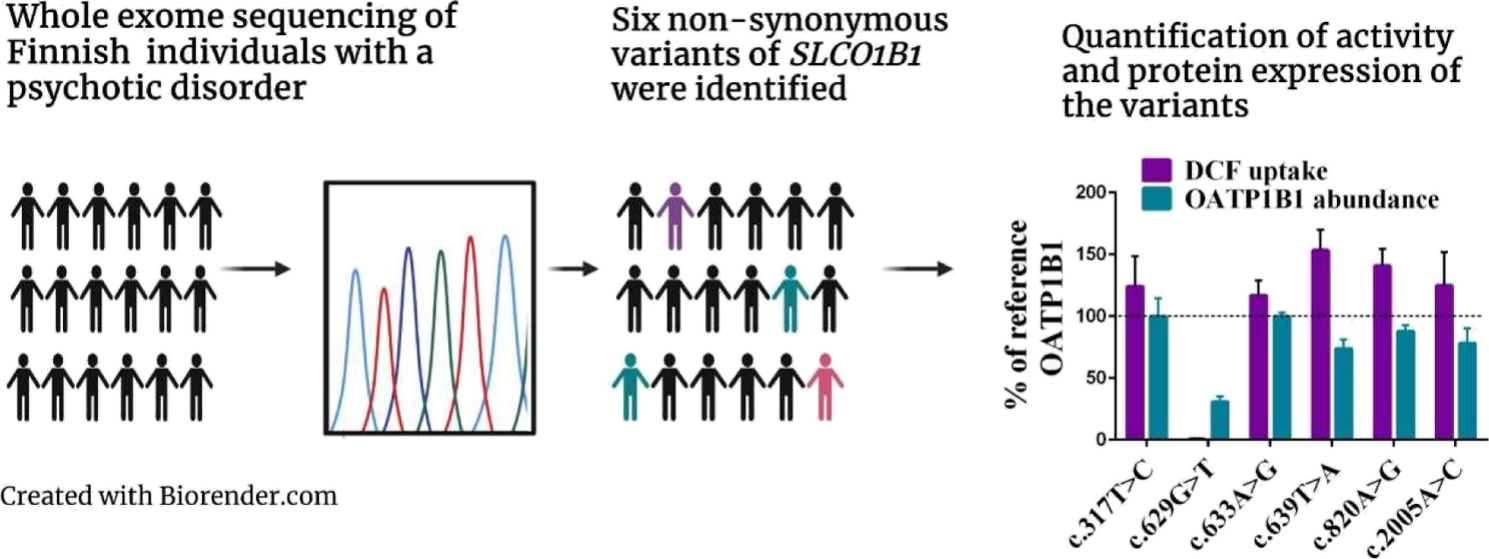

Variants in the *SLCO1B1* (solute carrier organic anion transporter family member 1B1) gene encoding the OATP1B1 (organic anion transporting polypeptide 1B1) protein are associated with altered transporter function that can predispose patients to adverse drug effects with statin treatment. We explored the effect of six rare *SLCO1B1* single nucleotide variants (SNVs) occurring in Finnish individuals with a psychotic disorder on expression and functionality of the OATP1B1 protein. The SUPER-Finland study has performed exome sequencing on 9381 individuals with at least one psychotic episode during their lifetime. *SLCO1B1* SNVs were annotated with PHRED-scaled combined annotation-dependent (CADD) scores and the Ensembl variant effect predictor. In vitro functionality studies were conducted for the SNVs with a PHRED-scaled CADD score of >10 and predicted to be missense. To estimate possible changes in transport activity caused by the variants, transport of 2′,7′-dichlorofluorescein (DCF) in OATP1B1-expressing HEK293 cells was measured. According to the findings, additional tests with rosuvastatin and estrone sulfate were conducted. The amount of OATP1B1 in crude membrane fractions was quantified using a liquid chromatography tandem mass spectrometry-based quantitative targeted absolute proteomics analysis. Six rare missense variants of *SLCO1B1* were identified in the study population, located in transmembrane helix 3: c.317T>C (p.106I>T), intracellular loop 2: c.629G>T (p.210G>V), c.633A>G (p.211I>M), c.639T>A (p.213N>L), transmembrane helix 6: 820A>G (p.274I>V), and the C-terminal end: 2005A>C (p.669N>H). Of these variants, *SLCO1B1* c.629G>T (p.210G>V) resulted in the loss of in vitro function, abolishing the uptake of DCF, estrone sulfate, and rosuvastatin and reducing the membrane protein expression to 31% of reference OATP1B1. Of the six rare missense variants, *SLCO1B1* c.629G>T (p.210G>V) causes a loss of function of OATP1B1 transport in vitro and severely decreases membrane protein abundance. Carriers of *SLCO1B1* c.629G>T might be susceptible to altered pharmacokinetics of OATP1B1 substrate drugs and might have increased likelihood of adverse drug effects such as statin-associated musculoskeletal symptoms.

## Introduction

Statins are among the most common prescription drugs in the world and are used for the reduction of low-density lipoprotein cholesterol concentration and prevention of cardiovascular disease. The solute carrier organic anion transporter family member 1B1 (*SLCO1B1*) gene encodes the organic anion transporting polypeptide 1B1 (OATP1B1), a transmembrane protein that is involved in the transport of drugs such as statins and other compounds from the blood into the hepatocytes.^1,2^

Single nucleotide variants (SNVs) in *SLCO1B1* are associated with impaired transporter function that can alter systemic exposure to statins. This can consequently predispose patients to adverse drug effects including statin-associated musculoskeletal symptoms (SAMS) and even rhabdomyolysis, impacting statin adherence and hindering the long-term effectiveness of statin therapy.^3−7^ Cardiovascular mortality is one of the leading causes of excess mortality in patients with schizophrenia.^8^ Antipsychotic medications, especially second-generation antipsychotic medications such as clozapine and olanzapine, can induce metabolic abnormalities including dyslipidemias.^9^ Therefore, statins are commonly prescribed to patients with psychotic disorders.

The U.S. Food and Drug Administration (FDA) and European Medicines Agency (EMA) acknowledge the significance of OATP1B1 variants in medication safety by providing guidelines for studying the drug transporter’s effect on pharmacokinetics in drug development.^10,11^

Pharmacogenetic research has suffered from small sample sizes, and finding a patient group of sufficient size is often challenging when exploring rare genetic variants.^12^ Rare variants of *SLCO1B1* are mainly unreported, although their effect on drug safety can be as consequential as the effect of common variants.^13,14^ Interpretation of genetic variants from vast amounts of whole exome sequencing data remains a challenge, and computational tools are needed to predict the functional impact of pharmacogenetic variants before the identified variants can be explored at a biological level.^15−17^ Various algorithms that are needed to predict the functional impacts of coding variants are available using standard criteria and scoring metrics or machine learning approaches. Biological assessment of in silico evaluated variants is necessary to generate clinically actionable recommendations.

We studied the potential clinical relevance of rare *SLCO1B1* SNVs occurring among 9381 Finnish individuals with a psychotic disorder recruited as part of the SUPER-Finland study. The variants were selected for in vitro expression and functionality experiments based on a damaging in silico prediction by the PHRED-scaled combined annotation-dependent (CADD) score algorithm^18,19^ and an Ensembl variant effect predictor (VEP)^20^ annotation of missense. Cellular uptake studies in OATP1B1-overexpressing HEK293 cells were conducted, and the amount of the OATP1B1 variant protein in crude membrane fraction HEK293 cells was quantified using a liquid chromatography tandem mass spectrometry (LC–MS/MS)-based quantitative targeted absolute proteomics (QTAP) analysis. Our results provide functional annotations for *SLCO1B1* c.317T>C (p.I106T), c.629G>T (p.G210V), c.633A>G (p.I211M), c.639T>A (p.N213K), c.820A>G (p.I274V), and c.2005A>C (p.N669H) variants.

## Material and Methods

### SUPER-Finland Study

The SUPER-Finland study recruited 10,474 participants aged >18 with a severe mental disorder from Finnish in- and outpatient psychiatric care, primary care, and housing units and additionally with newspaper advertising between the years 2016–2018. Subjects with a diagnosis of the schizophrenia spectrum disorder (ICD-10 codes F20 and F22–29), bipolar disorder (F30 and F31), or major depressive disorder with psychotic features (F32.3 and F33.3) were included in the study. Exclusion criteria were inability to give informed consent and age under 18 years. We put special focus on ensuring a wide coverage of known Finnish internal population subisolates.^21^ The SUPER-Finland study protocol will be described in a separate cohort profile manuscript that is in preparation.

DNA was extracted from participants’ blood samples collected by venipuncture (2× Vacutainer EDTA K2 5/4 mL, BD). A saliva sample (DNA OG-500, Oragene) was collected for DNA extraction when venipuncture was not possible. The samples were frozen (−20 °C) within 60 min of sampling and sent to the THL Biobank (Finnish Institute for Health and Welfare) within 3 months for long-term storage (−185 °C). A PerkinElmer Janus chemagic 360i Pro Workstation with the CMG-1074 kit was used to extract DNA from the EDTA blood tubes. Extraction of DNA from saliva samples (after incubation at + 50 °C, o/n) was performed by Chemagen Chemagic MSM I robot with the CMG-1035–1 kit. The DNA samples were genotyped and sequenced at the Broad Institute of MIT and Harvard, Boston Cambridge, Massachusetts, USA.

### Exome Sequencing of SUPER-Finland Study Samples

To discover rare *SLCO1B1* SNVs with possible changes in OATP1B1 protein function, we used exome data from 9381 SUPER-Finland study participants. Exome sequencing was carried out on the Illumina HiSeq platform using 151 base pair paired-end reads at the Broad Institute of Harvard and MIT. The samples were enriched with the Illumina Nextera capture kit and sequenced until 80% of the target capture was covered at 20×. The Picard sequence processing pipeline was used to process BAM files (http://broadinstitute.github.io/picard/), and the data were mapped to the human genome reference build 38 (GRCh38) using BWA.^22^ This procedure followed standard best practice alignment and read processing protocols as described earlier.^23,24^ Variants were called using the Genome Analysis Toolkit (GATK^25,26^). Local realignment around indels and recalibration of base qualities in each sample BAM were performed using GATK version 3.4. Each sample was called using HaplotypeCaller to create gVCF files containing every position of the exome with likelihoods for variants alleles or the genomic reference allele. All variants were annotated using the variant quality score recalibration (VQSR) tool in GATK version 3.6, resulting in a VCF with germline SNVs and indels for all samples used in the analyses. The variant joint calling corresponded to the pipeline used to create the GnomAD database.^23^

### In Silico Analysis of *SLCO1B1* Variants in the SUPER-Finland Study

The variants of chromosome 12 were converted to the Hail^27^ matrix table file format and annotated using CADD depletion^18,19^ (CADD, version 1.4/1.6) scores and with the VEP^20^ (version 95) tool through Hail (version 0.2, reference genome GRCh38). The variants were filtered using Hail to include only those having a call rate of > 0.99, a HWE *p*-value of > 1e-10, an allele count of > 1, a genotype quality of > 20, and a depth of > 10. The *SLCO1B1* variants with a PHRED-scaled CADD score of >10, predicted as missense variants by VEP and not included in the current CPIC guidelines for *SLCO1B1*, were selected for further in vitro expression and functional analyses.

### Preparation of Plasmids Carrying *SLCO1B1* Variants

Plasmids carrying the *SLCO1B1* variants were created as described by Kiander et al.^14^ The reference *SLCO1B1* gene used was Genebank accession number AJ132573.1, and the mutagenesis primers are described in Supporting Information Table S1. GATC Biotech’s (Constance, Germany) sequencing service confirmed the presence of the SNVs in the plasmids. Baculoviruses carrying the reference *SLCO1B1* gene, the variant *SLCO1B1* genes, and the previously cloned gene for enhanced yellow fluorescent protein (eYFP) as a negative control were produced as described earlier by Tikkanen et al.^28^

### Cell Culture and Protein Expression

HEK293 human kidney cells were cultured in Dulbecco’s modified Eagle medium (DMEM) and high-glucose GlutaMax culture medium supplemented with 10% fetal bovine serum (FBS) at 37 °C, 5% CO_2_. The cells (0.5 × 10^6^) were seeded in each well of 48-well plates (Thermo Fisher Scientific Nunc coated with poly-d-lysine in-house) 24 h prior to transduction with the baculoviruses. To stimulate the expression of proteins, sodium butyrate was added with the viruses at a final concentration of 5 mM (as per in-house optimization).

### Cellular Uptake Assays

The cellular uptake assay was performed 48 h post-transduction on a heated (37 °C) orbital shaker plate. For a 3 min preincubation, transport buffer (500 μL of HBSS with 4.17 mM NaHCO_3_ and 25 mM HEPES adjusted to a pH of 7.4 with NaOH) replaced the medium in the wells. After the buffer was removed, cellular uptake began when 125 μL of the test substrate solution was added into the wells. The uptake was stopped during the linear uptake phase by aspiration of the test solution (specific test times mentioned in the results). Three times wash with 500 μL of ice-cold transport buffer followed this step, and the cells were lysed with 125 μL of 0.1 M NaOH [2′,7′-dichlorofluorescein (DCF) and estrone sulfate] or 150 μL of a 3:1 methanol/water mixture (rosuvastatin samples). Fluorescence measurement (excitation 500 nm, emission 528 nm, and bandwidth 5 nm) using the multimode microplate reader Varioskan LUX (Thermo Fisher Scientific, Vantaa, Finland) of the cell lysates was performed to quantify the DCF. The [^3^H]-estrone sulfate-containing cell lysate was neutralized with equivalent moles of 1 M HCl before adding Optiphase HiSafe 3 scintillation liquid and measuring radioactivity of samples using a MicroBeta2 2450 Microplate Counter (PerkinElmer, Waltham, Massachusetts, USA). 10 μL of the cell lysate was mixed with 300 μL of the Coomassie Plus reagent and used for total protein amount quantification with absorbance analysis (595 nm) on Varioskan LUX. Rosuvastatin was quantified on a Sciex 5500Qtrap LC/MSMS system (ABSciex, Framingham, MA, USA) interfaced with an electrospray ionization (ESI) source as described previously.^29^

### Crude Membrane Extraction

Baculoviruses carrying either the reference or variant *SLCO1B1* and sodium butyrate (5 mM final concentration) were added to the HEK293 cells after the cells were cultured for 24 h in T175 flasks. After 48 h of culturing, the cells were pelleted by centrifugation (3000*g*, 15 min) and broken down using a Dounce tissue homogenizer and resuspended in Tris-sucrose (TS) buffer (10 mM Tris-HEPES, 250 mM sucrose, pH 7.4) while being kept on ice. The cell homogenate was centrifuged for 30 min (3220 g, 4 °C), separating the larger cellular organelles and the nucleus in the pellet. The resulting supernatant was subsequently separated into new tubes and centrifuged (21,000*g*, 4 °C, 99 min) again, resulting in a pellet containing the crude cell membrane. The protein sample was suspended in TS buffer, and the total protein concentration was quantified as previously described.

### LC–MS/MS-Based QTAP Analysis

The LC–MS/MS-based QTAP approach was used to quantify the absolute protein expression of OATP1B1 in the crude membrane preparations. The method for the protein sample preparation and quantitation using targeted LC–MS is described earlier.^14,30,31^ OATP1B1 and Na^+^/K^+^ ATPase signature peptides were quantified from 50 μg of crude membrane fractions. After denaturation and breakdown of the tertiary structure of the proteins, the crude membrane preparations were digested first with 1/100 LysC endopeptidase and after that with 1/100 TPCK-treated trypsin. A previously used isotope-labeled peptide mixture (3 fmol/μg protein) served as an internal standard.^14^ Quantification was conducted on a 6495 QQQ MS with a 1290 HPLC system and an AdvanceBio peptide Map Column, 2.7 μm, 2.1 × 250 mm (Agilent Technologies, Santa Clara, CA, USA) as described previously.^14,31^ The SNVs did not alter the amino acids in the analyzed peptide sequences. The peak area ratios of the analyte peptides and their respective internal standards were compared using the Skyline application (MacCoss Lab Software, Seattle, WA). The results of OATP1B1 expression are presented as relative to the Na^+^/K^+^–ATPase expression level and normalized to the reference OATP1B1 protein. The absolute amount of OATP1B1 in proteomics samples is presented in Supporting Information Table S2 and Figure S1.

### Data Analysis

The uptake of DCF was normalized to the total protein amount. The uptake into eYFP-expressing cells, representing passive influx, was subtracted from the uptake into OATP1B1-expressing cells, yielding active OATP1B1-mediated transport. The transport activity in OATP1B1 variant cells was then normalized to the cells expressing wild-type OATP1B1. The statistical significance of the differences in activity and expression levels was determined by one-way analysis of variance (ANOVA) with the Dunnett’s post hoc test for multiple comparisons (GraphPad Prism 6.05, GraphPad Software, San Diego, CA, USA). Extra-sum-of-squares F-test was used to assess the statistical significance of the observed changes in kinetic parameters of DCF transport using the same software.

### Ethics

The Coordinating Ethics Committee of the Hospital District of Helsinki and Uusimaa (HUS) gave a favorable ethics statement (202/13/03/00/15) for the SUPER-Finland study. Prior to inclusion, written informed consent was obtained and archived from each participant. Individual-level data were pseudonymized.

### Data and Code Availability

The computer code used in the analysis of this study is available from the corresponding author on reasonable request, and the SUPER-Finland study data are available from THL Biobank when released from the original study.

## Results

We evaluated the functionality of six SNVs of OATP1B1. The SNVs with their genomic positions, amino acid changes, and locations in OATP1B1 are shown in Table 1.

**Table 1:** Genomic Positions and Amino Acid Changes of the *SLCO1B1* SNVs

SNV^a^	variant ID^b^	position in GRCh38.p13^a^	amino acid change^b^
c.317T>C	rs200227560	chr12:21174667	Ile106Thr
c.629G>T	rs766417954	chr12:21178922	Gly210Val
c.633A>G	rs201722521	chr12:21178926	Ile211Met
c.639T>A	rs752897663	chr12:21178932	Asn213Lys
c.820A>G	rs762084290	chr12:21197038	Ile274Val
c.2005A>C	rs762293939	chr12:21239118	Asn669His

a Ensembl genome browser https://www.ensembl.org/index.html.

b dbSNP single nucleotide polymorphism database https://www.ncbi.nlm.nih.gov/snp/.

The variants were expressed in HEK293 cells, and the effect of the SNVs on transport activity was evaluated with a cellular uptake study using DCF as a substrate. An LC–MS/MS-based QTAP approach was used to quantify any changes in the protein abundance of OATP1B1 in crude membrane fractions.

### In Silico Predictions of *SLCO1B1* Variants in the SUPER-Finland Study

In silico predicted consequences of the six *SLCO1B1* variants are presented in Table 2, and allele frequencies of the variants are shown in Table 3.

**Table 2:** In Silico Prediction of Consequences of the *SLCO1B1* SNVs

SNV^a^	CADD PHRED score^b^	SIFT prediction^c^	PolyPhen prediction^d^	VEP consequence^e^
c.317T>C	10.860	0.7 (tolerated)	0.023 (benign)	missense
c.629G>T	23.400	0 (deleterious)	0.999 (probably damaging)	missense
c.633A>G	13.070	0.07 (tolerated)	0.821 (possibly damaging)	missense
c.639T>A	19.280	0.02 (deleterious)	0.038 (benign)	missense
c.820A>G	19.790	0.13 (tolerated)	0.277 (benign)	missense
c.2005A>C	23.000	0.02 (deleterious)	0.76 (possibly damaging)	missense

a Ensembl genome browser https://www.ensembl.org/index.html.

b PHRED-scaled CADD score through Hail.

c SIFT human protein online service https://sift.bii.a-star.edu.sg/ through Ensembl^a^.

d PolyPhen-2 online service for prediction of functional effects of human SNVs http://genetics.bwh.harvard.edu/pph2/ through Ensembl^a^.

e Ensembl VEP through Hail.

**Table 3:** Prevalence of the *SLCO1B1* SNVs

SNV^a^	allele count in SUPER	number of carriers in SUPER	frequency in SUPER	Finnish frequency^b^	non-Finnish European frequency^b^	global frequency^b^
c.317T>C	4	4	2.1 × 10^–4^	4.6 × 10^–5^	7.4 × 10^–4^	7.0 × 10^–4^
c.629G>T	2	2	1.1 × 10^–4^	2.3 × 10^–4^	0.0	3.2 × 10^–5^
c.633A>G	2	2	1.1 × 10^–4^	0.0	8.8 × 10^–6^	3.6 × 10^–3^
c.639T>A	16	16	8.5 × 10^–4^	8.9 × 10^–4^	0.0	7.6 × 10^–5^
c.820A>G	2	2	1.1 × 10^–4^	4.6 × 10^–5^	0.0	4.0 × 10^–6^
c.2005A>C	2	2	1.1 × 10^–4^	2.3 × 10^–4^	0.0	2.0 × 10^–5^

a Ensembl genome browser https://www.ensembl.org/index.html.

b Exome population frequencies are retrieved from the gnomAD browser version 2.1.1 https://gnomad.broadinstitute.org/. Allele frequencies are counted by dividing the allele count by the overall number of alleles in a population.

**Figure 1:**
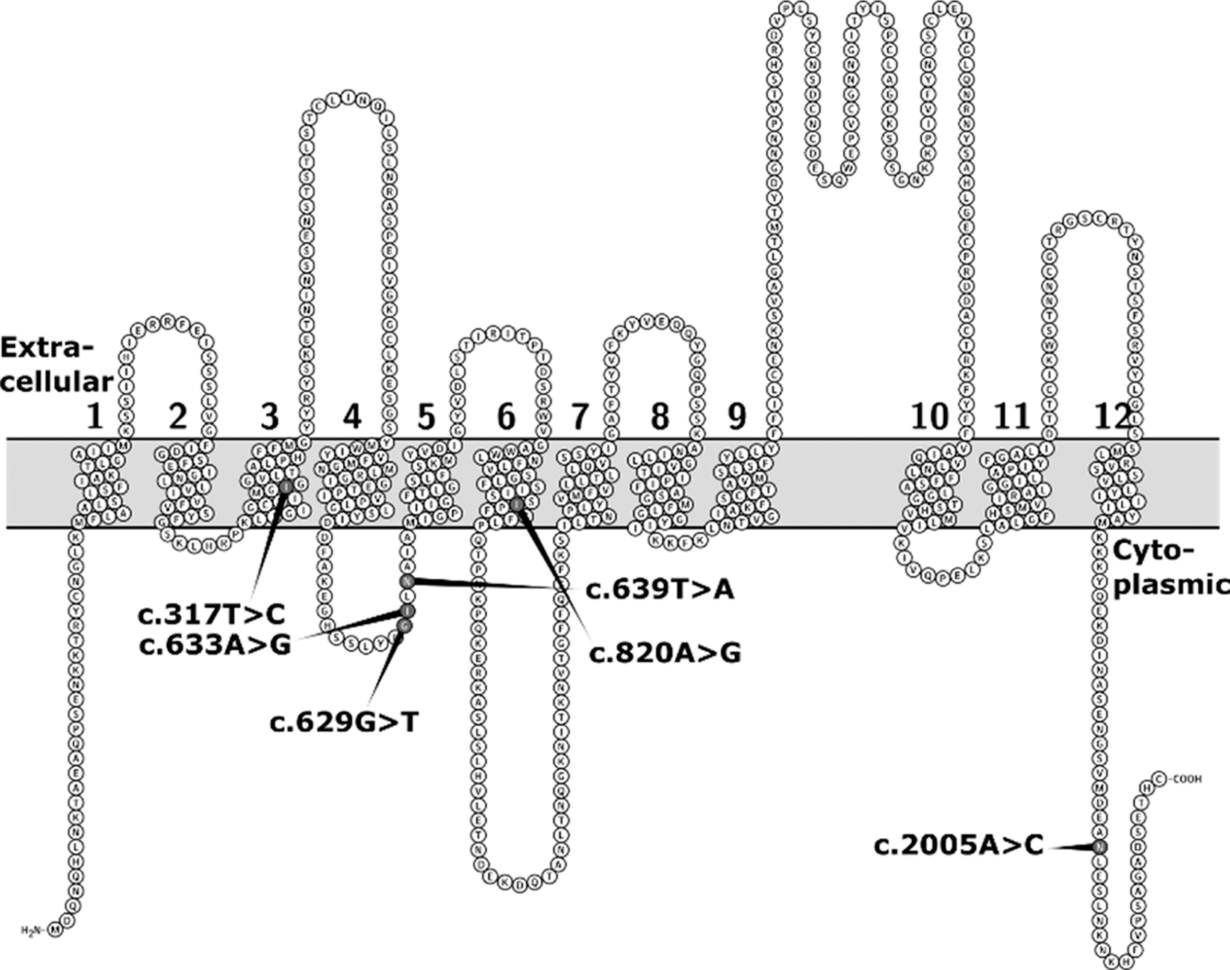
Location of the amino acids affected by the identified *SLCO1B1* SNVs in two-dimensional prediction of the OATP1B1 transporter. Numbers 1–12 denote the putative transmembrane helices. The figure is based on the Uniprot entry Q9Y6L6 and generated using Protter.^32^

SUPER-Finland subjects are heterozygous for the studied variants. The most frequent SNVs were c.639T>A, which was found in 16 subjects, and c.317T>C, which was found in four subjects. The other four SNVs were found in only two subjects. Based on the in silico predictions by CADD, SIFT, and PolyPhen, c.629G>T and c.2005A>C were most likely to have a damaging effect, and c.317T>C was most likely to be tolerated, while the predictions on the three other variants were more conflicting. All the variants are predicted to be missense variants by VEP. Two-dimensional prediction of the OATP1B1 transporter with the amino acid locations of the identified variants is presented in Figure 1.

### In Vitro Transport Activity and Membrane Protein Expression

**Figure 2:**
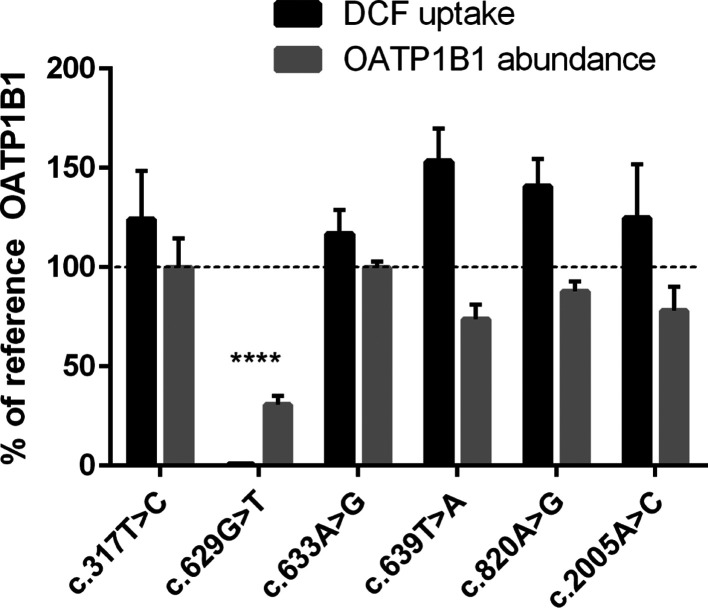
OATP1B1-mediated transport of 1 μM DCF into HEK293 cells over 15 min: results are calculated as the mean of four experiments conducted in quadruplicates and represented % of reference OATP1B1 transport ± SEM (*n* = 4). LC–MS/MS proteomics analysis of 50 μg of HEK293 crude membrane preparations expressing variant OATP1B1: abundance of OATP1B1 was quantified in four independent samples, normalized to Na^+^/K^+^-ATPase and reference OATP1B1 abundance (100%). The results are presented as mean ± SEM (*n* = 4). **** = *P* < 0.0001 (compared to the reference) ANOVA + Dunnett’s post hoc test.

**Figure 3:**
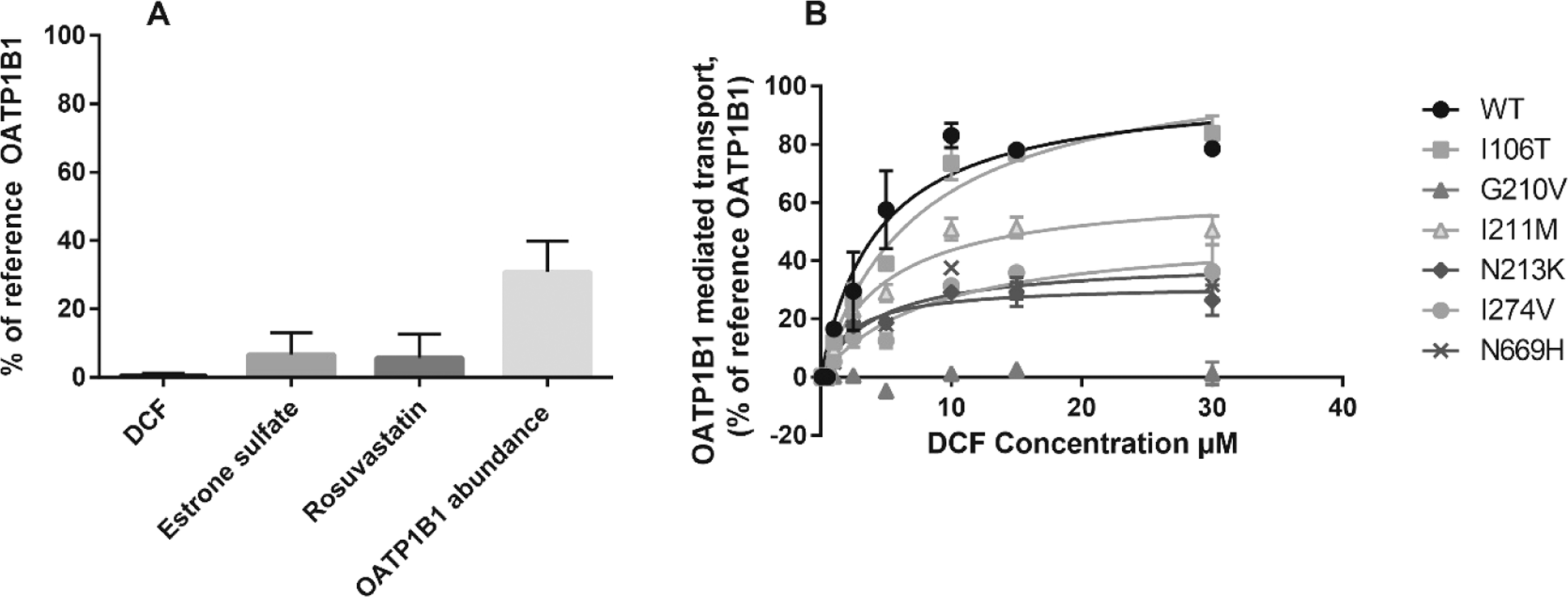
A: OATP1B1-mediated transport of 1 μM DCF (15 min), 0.5 μM estrone sulfate (2 min), and 5 μM (2 min) rosuvastatin into SLCO1B1 c.629G>T (p.G210V)-expressing HEK292 cells. Results are calculated as DCF: mean of four experiments conducted in quadruplicates and presented as % of reference OATP1B1 transport ± SEM (*n* = 4), estrone sulfate, and rosuvastatin: a single experiment, three technical replicates presented as % of reference OATP1B1 transport ± SD (*n* = 3). LC–MS/MS proteomics: four independent samples, normalized to Na^+^/K^+^-ATPase and reference OATP1B1 abundance (100%). B: concentration dependency assay of DCF over 15 min, single assay with two technical replicates.

We discovered that c.629G>T (p.G210V) abolishes the transport activity of OATP1B1 (Figures 2 and 3). Membrane protein abundance was also significantly decreased by approximately 70% compared to the OATP1B1 reference. The other variants did not alter the transport activity in the single concentration (1 μM DCF) assays or membrane protein expression to a statistically significant degree (Figure 2). However, in a concentration dependency assay, several variants showed either altered affinity (*K*_m_) to the transporter or altered maximum velocity (*V*_max_) (Figure 3 and Table 4).

**Table 4:** Abundance Data Are Normalized to the Reference and Also Presented in Figure 2^a^

variant	DCF *K*_m_ (μM)	(95% CI)	DCF *V*_max_ (% reference)	(95% CI)	OATP1B1 abundance (% WT) ± SEM
reference	4.3	1.8 to 6.9	100	81.5 to 118.8	
I106T	7.4**	4.4 to 10.5	111.2	93.8 to 128.5	99.8 ± 14.6
I211M	4.8	2.5 to 7.2	64.6****	54.3 to 75	99.8 ± 3.1
N213K	2.3*	0.8 to 3.9	31.8****	26.3 to 37.2	73.8 ± 7.6
I274V	8.4*	2.5 to 14.3	50.5***	36.4 to 64.5	87.8 ± 5.1
N669H	4.2	1.2 to 7.2	40.1****	31.1 to 49.1	78 ± 12.3

a Maximum velocity of transport (*V*_max_) of the tested variants is normalized to the calculated *V*_max_ of WT OATP1B1 from a single experiment with two replicates. Curve fitting was not possible for variant G210V due to the lack of sufficient uptake. **** = *P* ≤ 0.0001, *** = *P* ≤ 0.001, ** = *P* ≤ 0.01, and * = *P* ≤ 0.05 according to the extra-sum-of-squares F-test.

Interestingly, however, c.639T>A (p.213N>K) seemed to enhance transport activity at a low concentration (1 μM) of DCF, which was on average 162% of reference OATP1B1 (Figure 2). The concentration dependency assay revealed that this variant increases DCF affinity to OATP1B1, evident from the decreased *K*_m_ parameter, but reduced the *V*_max_ (Table 4). The protein expression of c.639T>A (p.213N>K), however, was not increased.

## Discussion

We have provided novel functional annotations for six *SLCO1B1* variants. Among these, c.629G>T (p.210G>V) was found to be a loss-of-function variant in vitro. According to GnomAD,^23^ the variant has been detected only in Finnish and South Asian populations. We used computational algorithms to prioritize *SLCO1B1* variants for in vitro validation. The PHRED-scaled CADD score for this variant was 23.4, and it was predicted to be deleterious by SIFT and probably damaging by PolyPhen. A variant with a similar CADD score of 23.0 (c.2005A>C and p.N669H) and classified as deleterious and possibly damaging by SIFT and PolyPhen, respectively, had normal function according to our in vitro studies. This underlines the difficulty of computational functional annotation of missense variants. Due to the enormous number of variants in whole exome sequencing studies, these studies need to rely on computational functional predictions as not every potential loss-of-function variant throughout the genome can be tested in the laboratory. The definition of a computationally predicted loss-of-function variant is a matter of sensitivity and specificity. Selecting a conservative definition would filter out potential disease-causing mutations, while too liberal a threshold creates noise in association signals. As the CADD score is based on the regional mutational constraint,^18,19^ it might not be optimal in capturing pharmacogenetic variants as genes responsible for the metabolism and transport of xenobiotic compounds might not be under as strong selection as genes related to, for example, brain development.

*SLCO1B1* c.629G>T results in the replacement of a glycine with a valine in position 210. Glycine has no side chain and has a very high tendency to build turns in the secondary structure of polypeptide chains.^33^ Valine, on the other hand, has a preference to form strands and obstructs the formation of turns and bends in the secondary structure. Thus, it is understandable that this substitution could impair the function of OATP1B1. Indeed, our results show that *SLCO1B1* c.629G>T reduces the uptake of three different substrates to less than 10% of the reference (Figure 3A). *SLCO1B1* c.317T>C (p.106I>T), c.633A>G (p.211I>M), and c.820A>G (p.274I>V) are all in codons that originally code for isoleucine, but the SNVs result in substitutions with amino acids with similar properties. Nevertheless, all of these variants reduced the *V*_max_ of DCF uptake significantly (Table 4). *SLCO1B1* c.639T>A (p.213N>K) changes an amidic asparagine into a basic lysine, which in physiological pH has a positive charge. While the actual three-dimensional structure of the OATP proteins remain unknown, OATPs are predicted to form a positively charged pore.^34^ Since many OATP1B1 substrates are anionic compounds, an additional positive charge near this putative pore in the intracellular loop where c.639T>A (p.N213K) is located might increase substrate affinity. Indeed, the *K*_m_ value for DCF transport was significantly decreased (Table 4). According to GnomAD,^23^ the c.639T>A variant has been identified only in Finnish population. A similar gain of charge occurs in *SLCO1B1* c.1007C>G (p.P336R, located in TMH 7) and was shown in vitro to have substrate-dependent normal to enhanced OATP1B1 transport activity.^14,35^ Likewise, the effect of c.639T>A may be substrate-dependent, which needs to be clarified in future research. On the other hand, while *SLCO1B1* c.2005A>C (p.669N>H) also gains a charge, it decreases *V*_max_ of DCF transport but does not alter affinity (*K*_m_) or activity at a low DCF concentration (Figures 2 and 3B and Table 4). This could suggest that this amino acid position is not as important for substrate recognition of DCF transport. While the crude membrane fractions do not exclude intracellular membranes such as the endoplasmic reticulum^36^ when comparing the immunofluorescence images and proteomics results, decreased abundance in proteomics samples appears to be paired with reduced plasma membrane localization.^14^ Nonetheless, additional studies of, e.g., biotinylated samples could help confirm these findings. All things considered, these findings provide additional insights into amino acids critical to OATP1B1 function and suggest that c.629G>T (p.210G>V) could be characterized as a loss-of-function variant while the other variants are most likely clinically benign.

Polypharmacy is common in patients treated with antipsychotic medication, leading to a high probability of adverse drug effects and drug–drug interactions^37^ that an unfavorable genotype might complicate. While, to date, according to The University of Washington Drug Interaction Database (DIDB),^38^ no central nervous system agents have been identified as OATP1B1 substrates, many other drugs can be prescribed to these patients to treat their somatic diseases. Among these are often statins to treat dyslipidemias, which are associated with antipsychotic medications.^9^ The association between statin use and muscle symptoms has been thoroughly investigated. Variants in *SLCO1B1* are associated with myopathy in particular when there is a documented increase in creatinine kinase concentration in blood.^3,39^ A poor function phenotype of OATP1B1 can increase the probability for adverse effects during statin treatment, thus reducing medication adherence.

The SLCO1B1*5 and SLCO1B1*15 haplotypes that contain the function-impairing c.521T>C SNV are associated with reduced hepatic clearance of substrate drugs, increased systemic exposure, and increased risk of SAMS.^1,6,7^ The current gene-based prescribing guideline from the Clinical Pharmacogenetics Implementation Consortium (CPIC) states that individuals with decreased and poor function phenotypes of OATP1B1 should limit statin doses and those with poor function phenotypes should avoid simvastatin altogether.^6^ Based on our in vitro studies, c.629G>T (p.G210G>V) could be categorized as a poor function phenotype since the in vitro activity and abundance is comparable to that of the *SLCO1B1* c.521T>C genotype.^1,14^ However, homozygous carriers of a rare variant like c.629G>T (p.G210G>V) are very uncommon. It is more likely to occur in a heterozygous state, as observed in our study population (Table 3), or, like c.521T>C in SLCO1B1*15, in combination with c.388A>G, which has a frequency of approximately 26–77% depending on the population.^40^ Additionally, the c.521T>C allele is quite common in certain populations, for example, the minor allele frequency in Finnish Europeans is 0.21.^23^ Consequently, even as many as every fourth c.629G>T carrier is likely to carry c.521T>C, resulting in a poor function phenotype. While the heterozygous genotype would limit the clinical consequences from a poor function phenotype to a decreased function phenotype, these individuals would still have an increased risk of SAMS during high-dose statin treatment and thus could benefit from genotype-guided dosing.

## Conclusions

We have identified a novel *SLCO1B1* loss-of-function mutation, which could be accounted for in guidelines describing individualized lipid-lowering therapy. The variant c.629G>T (p.210G>V) abolishes the transport of all three of the tested OATP1B1 substrates, suggesting that it might predispose patients to an increased risk of SAMS during statin treatment. This emphasizes the need to include diverse populations in drug trials as adverse effects related to population-specific variants might otherwise be missed. While the kinetic parameters of DCF transport were altered by several of the other tested variants, *SLCO1B1* variants c.317T>C, c.633A>G, c.639T>A, c.820A>G, and c.2005A>C could be considered normal-function variants.

## Supporting Information Available

Mutagenesis primers used in creating SNVs in the *SLCO1B1* gene, the absolute amount of OATP1B1 in proteomics samples, and the absolute amount of OATP1B1 and Na^+^/K^+^/ATPase in proteomics samples (PDF)
