# Supplementary material for: Functional Characterization of Six SLCO1B1 (OATP1B1) Variants Observed in Finnish Individuals with a Psychotic Disorder
Source: Mol Pharm. 2023 Feb 13;20(3):1500–8. doi: 10.1021/acs.molpharmaceut.2c00715 (PMC9996821; doi:10.1021/acs.molpharmaceut.2c00715)

Supplementary Table I Mutagenesis primers used in creating SNVs in the SLCO1B1 gene. Small case letter denotes the nucleotide substitution.

SNV	Amino acid change	Forward	Reverse
c.317T>C	Ile106Thr	ATTATGGGAAcTGGAGGTGTT	GAAACAACCGATTCCAATTAAC
c.629G>T	Gly210Val	ATTTAGGTATgTTGAATGCAATAG	ACAAAGAAGAATGTCCTTC
c.633A>G	Ile211Met	TTGTATTTAGtTATATTGAATGCAATAG	AGAAGAATGTCCTTCTTTAG
c.639T>A	Asn213Lys	GTATATTGAAaGCAATAGCAATGATTG	CTAAATACAAAGAAGAATGTCC
c.820A>G	Ile274Val	TATTTCTTCCgTACCATTCTTTTTC	ATGGAGAATAGTCCAGAC
c.2005A>C	Asn669His	GGATGAAGCAcACTTAGAATCC	ATGACACTTCCATTTTCTG

Supplementary Table II Absolute amount of OATP1B1 in proteomics samples.
Na^+/K^+/ATPase unique peptide: AAVPDAVGK, OATP1B1 unique peptide: LNTVGIAK

Variant	Average OATP1B1 abundance fmol/µg protein	Standard error of the mean	Average Na^+/K^+/ATPase abundance fmol/µg protein	Standard error of the mean
Reference	1.09	0.52	2.45	0.59
I106T	1.09	0.49	2.61	0.58
G210V	0.21	0.059	1.81	0.27
I211M	0.95	0.27	2.49	0.51
N213K	0.63	0.29	1.84	0.23
I274V	0.73	0.29	1.92	0.14
N669H	0.62	0.23	1.92	0.36

Supplementary Figure 1 Absolute amount of OATP1B1 and Na^+/K^+/ATPase in proteomics samples: individual samples coupling the abundance of two unique peptides AAVPDAVGK for Na^+/K^+/ATPase and LNTVGIAK for OATP1B1.

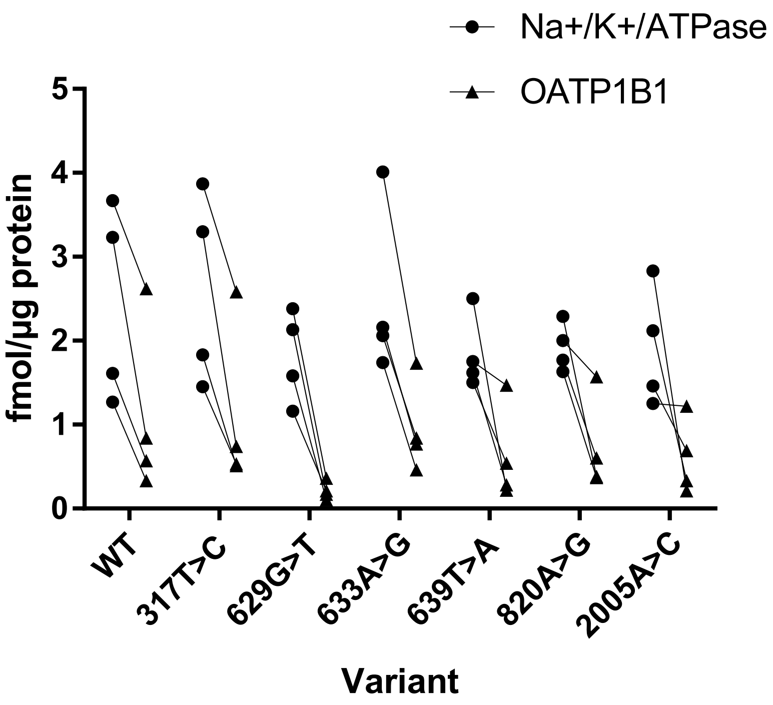

Supplement: Supplementary file 1 — mp2c00715_si_001.pdf [file mp2c00715_si_001.pdf]
